# Editorial: Plant Thermodynamics

**DOI:** 10.3389/fpls.2022.932245

**Published:** 2022-06-15

**Authors:** Bartolome Sabater, Keith R. Skene

**Affiliations:** ^1^Department Ciencias de la Vida (Fisiología Vegetal), University of Alcalá, Alcalá de Henares, Spain; ^2^Biosphere Research Institute, Angus, United Kingdom

**Keywords:** energetics, enthalpy, entropy, free energy, nutrient absorption, photosynthesis, respiration

The general laws of Thermodynamics provide the bases for our comprehension of energy conversions in the Universe, including living beings and their interactions with their environments. Through photosynthesis, plants carry out the key step of the conversion of radiant energy to chemically storable energy. Plant transpiration dissipates a variable, but significant, fraction of incident radiant energy. In addition, general metabolism, and plant-specific processes such as nutrient transport, involve energy conversions which impact upon plant functionality, ecology, and environment.

Despite some initial polemics on the validity of the second thermodynamic principle in terms of the conversion of radiant to chemical energy in photosynthesis, the core importance of thermodynamics has now been demonstrated in all plant processes and this has allowed testing of proposed functional models and the development of tools to understand the processes involved. Therefore, thermodynamic approaches are implicit or explicit in many plant investigations. In addition to the most frequently considered thermodynamic functions, free energy and enthalpy, the entropy function is receiving increasing specific attention to understand environmental and evolutionary processes.

The four articles in this volume topic are representative of the wide diversity of processes whose understanding benefits from thermodynamic approaches.

The isothermal kinetics of solute absorption by roots are complex and frequently show abrupt changes of V_max_ and affinity in a narrow range of concentrations. The mechanism of that multiphasic uptake of nutrient by roots is a long-standing molecular question with obvious relevance in agronomy and soil ecology. Within this *Plant Thermodynamics* topic, a non-linear dynamics approach (Deunff et al.) has been reported to explain the bifurcation of the nitrate uptake by roots of *Brassica napus* around external 0.28 mM nitrate concentrations. Contradictory evidence on the involvement of two different transporters and the rates of influx and efflux would appear to be resolved through a non-linear dynamics approach suggesting the involvement of only one transporter (BnNPF6/NRT1.1) subjected to allosteric regulation between low and high nitrate concentrations.

The rapid responses of leaf respiration to temperature changes have been investigated by an empirical model (the Kavanau model) and a mechanistic model (Macromolecular Rate Theory, MMRT) in five species of urban subtropical trees (Xu et al.). The MMRT model allows an easy determination of enthalpy, entropy, and Gibbs free energy of activation for plant respiration. The results show differences above 42°C among the five species for the effect of temperature change that could be used to improve predictions of the effects of climate change on plant respiration and for the selection of adapted trees.

The standard enthalpy of formation, standard molar entropy, standard Gibbs free energy of formation and standard molar heat capacity have been calculated from the empirical formulas of cultured cotton, rice, bean, sugar cane, and corn and from their growth stoichiometry (Popovic et al.). The concept of a driving force (Gibbs energy) has been generalized to the usable photosynthetic energy (−2.3 MJ/C-mol average) and related to growth rates by determining phenomenological L coefficients (taking into account the kinetic factors). The approach allows a new analysis of the factors that influence plant growth. Depending on plant species and environmental conditions, phenomenological coefficients differ within two orders of magnitude range. Bean shows the lowest L coefficients as expected from its additional energy requirements for nitrogen fixation.

The increase of the efficiency of the photosynthetic light conversion is key to improving crop yields. Specifically focussing on field grown wheat, the diurnal and seasonal variation of the efficiency of energy conversion has been investigated (Song et al.) utilizing the gas exchange method in plants with different canopy architecture and high temporal resolution along day and season. Efficiency varied between 0.01 and 0.05 mol CO_2_ mol^−1^ photon, being the highest at medium PAR. As season-averaged, 1 MJ radiation yields around 1.6 g biomass. The great variation of efficiency when measured at high temporal resolution suggests that the harvest yield of wheat would increase through potential improvements of canopy photosynthesis.

Without doubt, similar investigations on plant thermodynamics will continue yielding significant and important knowledge on the path from basic to applied science and on environmental issues.

## Author Contributions

All authors listed have made a substantial, direct, and intellectual contribution to the work and approved it for publication.

## Conflict of Interest

The authors declare that the research was conducted in the absence of any commercial or financial relationships that could be construed as a potential conflict of interest.

## Publisher's Note

All claims expressed in this article are solely those of the authors and do not necessarily represent those of their affiliated organizations, or those of the publisher, the editors and the reviewers. Any product that may be evaluated in this article, or claim that may be made by its manufacturer, is not guaranteed or endorsed by the publisher.

